# Lamotrigine Attenuates Lipopolysaccharide-Induced Working-Memory Impairment and Is Associated with Changes in Neuroinflammatory, Oxidative-Stress, and Apoptosis-Related Markers

**DOI:** 10.3390/brainsci16090971

**Published:** 2026-09-14

**Authors:** Bashair Hamed Alharbi, Abdulaziz Arif A. Alshammari, Vasudevan Mani

**Affiliations:** Department of Pharmacology and Toxicology, College of Pharmacy, Qassim University, Buraydah 51452, Saudi Arabia; 451214229@qu.edu.sa (B.H.A.); aziz.alhmzani@gmail.com (A.A.A.A.)

**Keywords:** apoptosis, lamotrigine, lipopolysaccharide, memory impairment, neuroinflammation, oxidative stress

## Abstract

**Highlights:**

**What are the main findings?**

- Lamotrigine (LTG) ameliorated lipopolysaccharide (LPS)-induced working-memory impairment and suppressed neuroinflammatory responses in the prefrontal cortex.
- LTG reduced oxidative stress and pro-apoptotic signaling while restoring endogenous antioxidant and anti-apoptotic defenses.

**What are the implications of the main findings?**

- LTG may exert neuroprotective effects through coordinated regulation of inflammatory, oxidative, and apoptotic pathways.
- These findings support further investigation of LTG as a potential therapeutic strategy for inflammation-associated working-memory impairment.

**Abstract:**

Background/Objectives: Persistent neuroinflammation, oxidative imbalance, and neuronal apoptosis are key mechanisms underlying memory loss and working-memory impairment in many neurodegenerative conditions. LTG, an established anticonvulsant recognized for its glutamate-modulating and immune-dampening actions, has been reported to exert neuroprotective actions beyond seizure control. This study investigated whether LTG can counteract LPS-induced deficits in cognition and neuroinflammatory responses in rats by modulating oxidative stress- and apoptosis-related markers. Methods: A total of twenty-four rats were allocated into four different groups: Control, LPS, LTG, and LTG + LPS. LTG (30 mg/kg, orally) was administered for 15 days, whereas LPS (1 mg/kg, intraperitoneally) was administered during the final four days to provoke neuroinflammation. Working memory abilities were assessed utilizing the Y-maze spontaneous alternation test. Subsequently, brain tissues were analyzed for inflammatory mediators (COX-2, TNF-α, IL-6), apoptotic markers (Caspase-3, Bcl-2, BAX), and oxidative stress indicators (MDA, GSH, catalase). Results: LPS exposure produced significant memory deterioration, accompanied by elevated COX-2, TNF-α, IL-6, and MDA levels, along with reduced GSH and catalase activity, indicating oxidative and inflammatory stress. BAX and Caspase-3 expression were markedly enhanced, whereas Bcl-2 expression was significantly reduced. Pretreatment with LTG markedly attenuated the LPS-induced decline in Y-maze performance, cytokine elevation, and alterations in redox and apoptotic parameters. Conclusions: LTG effectively attenuated LPS-induced cognitive impairment and neuroinflammatory responses, accompanied by improvements in oxidative stress- and apoptosis-related markers. These findings support further investigation of the potential preventive or neuroprotective effects of LTG against neuroinflammation-associated working-memory impairment.

## 1. Introduction

**Figure 1:**
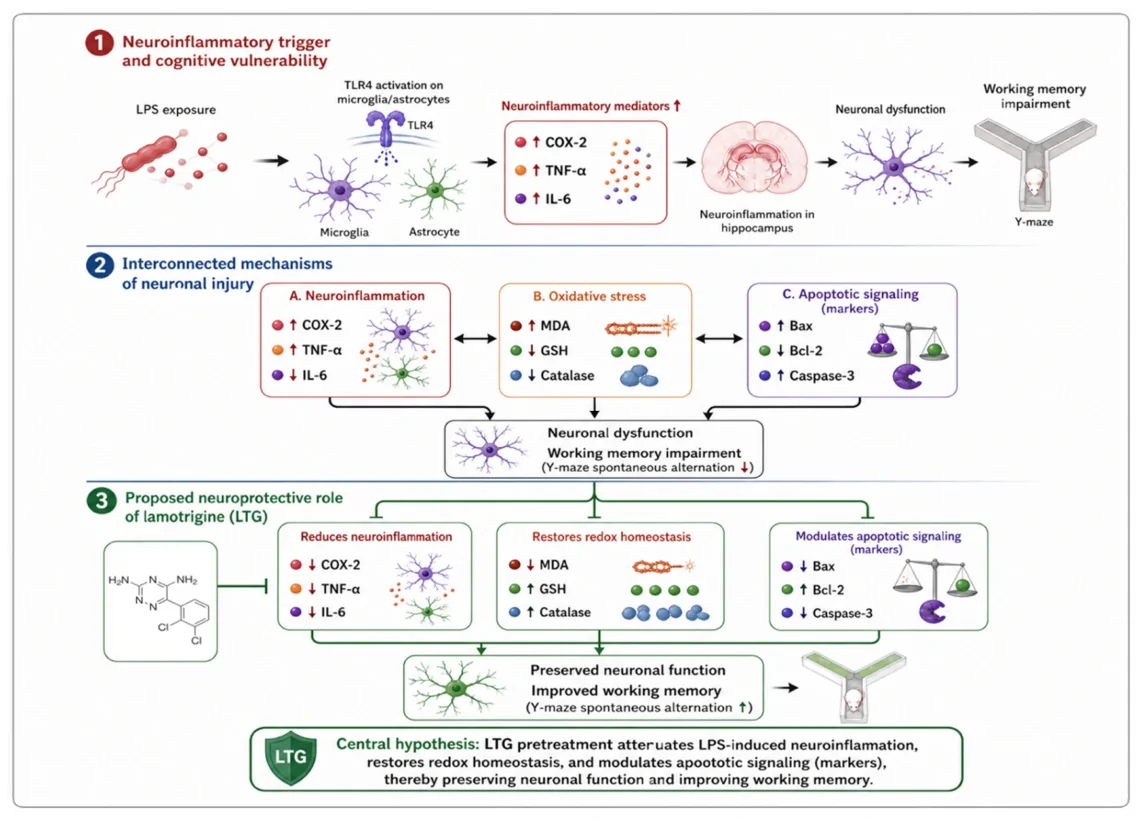
Proposed mechanisms of LPS-induced neurotoxicity and the neuroprotective effects of lamotrigine (LTG). LPS induces neuroinflammation, oxidative stress, and apoptotic signaling, leading to neuronal dysfunction and impaired working memory. LTG is proposed to attenuate these mechanisms by reducing neuroinflammation, restoring redox homeostasis, and modulating apoptotic signaling, thereby preserving neuronal function and improving memory performance.

The nervous system is particularly vulnerable to chemical- and drug-induced injury because of its high metabolic demands, complex cellular architecture, and limited regenerative capacity. Consequently, neurotoxicity can disrupt neuronal homeostasis and cognitive function through interconnected mechanisms, including neuroinflammation, oxidative stress, mitochondrial dysfunction, and apoptotic signaling [1]. Data from several population groups link higher circulating inflammatory markers to weaker neurobehavioral and cognitive outcomes, an association documented both in older adults with early cognitive decline and in patients with major psychiatric illness [2]. Inflammatory signals originating outside the brain can still reach the CNS and interfere with neuronal processes such as synaptic remodeling and the generation of new neurons, ultimately undermining cognitive performance [3]. Cytokines such as IL-1, IL-6, and TNF-α are repeatedly cited as drivers of inflammation-linked cognitive impairment. Dysregulation of these mediators can alter neuronal function and contribute to impairments in learning, memory, and neurobehavioral performance [4]. However, biomarker levels may also be influenced by factors unrelated to neurotoxicity, including age, systemic inflammation, and renal function, highlighting the need for careful interpretation of biomarker changes within their broader biological context [5]. Working memory underlies higher-order cognition, briefly holding and manipulating information needed for reasoning, problem solving, learning, and decision-making. Its efficiency depends on the functional integrity of neural networks within the prefrontal cortex, and disruption of these networks by injury or persistent inflammation can impair working memory and executive control [6,7,8,9] (Figure 1).

Several interconnected biological processes contribute to inflammation-associated cognitive decline, notably neuroinflammation, oxidative stress, and apoptosis, each capable of damaging neuronal structure and function [10,11]. When microglia and astrocytes remain chronically activated during inflammation, they release excess amounts of cytokines, including TNF-α and IL-6 [12]. A key downstream mediator of this inflammatory cascade is cyclooxygenase-2 (COX-2), an inducible enzyme that generates pro-inflammatory prostaglandins and amplifies neuroinflammatory responses. Heightened COX-2 activity has repeatedly been linked to excitotoxic injury, oxidative damage, lipid peroxidation, and greater neuronal susceptibility to harm [13]. Oxidative stress further damages cellular membranes, proteins, and DNA, while disruption of the balance between pro-apoptotic BAX and anti-apoptotic Bcl-2 promotes mitochondrial apoptotic signaling and Caspase-3 activation [10,14,15,16]. Considered jointly, Caspase-3, Bcl-2, and BAX offer a composite readout of the apoptotic cascade that can help gauge whether a drug is neurotoxic or neuroprotective [17,18]. These interacting pathways may contribute to neuronal dysfunction and subsequent cognitive deterioration. Accordingly, pharmacological strategies targeting oxidative stress, mitochondrial dysfunction, excitotoxicity, inflammation, and apoptosis represent direct and clinically relevant approaches to mitigating chemical- and drug-induced neurotoxicity [19,20,21]. An effective neuroprotective strategy is expected to do more than ease downstream symptoms; it should safeguard neuronal architecture and function from the earliest phase of the insult [22]. Given the central role of oxidative stress in toxic injury, antioxidant-based interventions have also emerged as promising neuroprotective strategies [23] (Figure 1).

As a preclinical tool, the LPS neuroinflammation paradigm is commonly used to probe how inflammation drives cognitive impairment and its underlying mechanisms [24]. LPS, a bacterial endotoxin derived from the cell wall of Gram-negative bacteria, activates Toll-like receptor 4-dependent inflammatory signaling and elicits robust inflammatory and oxidative responses. Peripheral or intracerebral LPS administration has repeatedly been reported to impair spatial learning, recognition memory, and working memory, making this model useful for evaluating potential neuroprotective interventions [24,25]. Pharmacological modulation of neuroinflammation can attenuate inflammation-associated behavioral deficits and normalize pro-inflammatory biomarkers [26]. Among potential neuroprotective agents, beyond its established role as an anticonvulsant and mood stabilizer, LTG is increasingly recognized for potential neuroprotective activity [27,28,29,30]. Previous research from our group demonstrated that psychotropic agents, including aripiprazole and betahistine, can counteract LPS-induced neurotoxicity through multimodal anti-inflammatory and anti-apoptotic mechanisms [25,31].

Direct evidence linking LTG to LPS-induced neurotoxicity remains scarce and largely confined to peripheral or in vitro settings. Abu-rish et al. demonstrated that LTG suppresses basal and LPS-induced IL-6 and TNF-α secretion, both in vivo in mice and in vitro in peritoneal macrophages and RAW264.7 cells; LPS-induced IL-1β secretion was also significantly suppressed in RAW264.7 cells, although in vivo LTG had no significant effect on LPS-induced IL-1β [32]. A related report showed that lamotrigine reverses the pro-inflammatory cytokine release triggered by quetiapine in LPS-stimulated microglial cell cultures, whereas combining lamotrigine with the classical mood stabilizers lithium or valproate alone tended, instead, to increase cytokine release [33]. Neither study, however, examined central nervous system outcomes in vivo or assessed cognitive performance. Separately, the neuroprotective and cognitive-enhancing properties of LTG have been reported in non-LPS models of neurotoxicity: LTG improved executive function and reduced inflammatory markers in an amyloid-based Alzheimer’s-like mouse model [34], preserved cognitive performance and limited synaptic and neuronal damage in a transgenic Alzheimer’s disease model [35], attenuated oxidative-nitrosative stress alongside spatial learning deficits following global cerebral ischemia [36], and produced region-specific improvements in oxidative markers and anhedonic behavior in a chronic mild stress model, albeit alongside increased lipid peroxidation in the prefrontal cortex and amygdala of non-stressed animals [33]. None of these studies, however, employed the LPS-induced neuroinflammation paradigm, which provides a more selective and reproducible way to isolate inflammation-driven cognitive dysfunction from amyloid- or ischemia-driven injury. Consequently, whether LTG can protect against LPS-induced working-memory impairment, and whether such protection is associated with concurrent changes across neuroinflammatory, oxidative, and apoptotic pathways within the same in vivo experimental model, remains insufficiently characterized.

While LTG’s anti-inflammatory and antioxidant actions are documented individually, no study has yet examined its impact on LPS-induced working-memory deficits alongside a combined inflammatory, oxidative, and apoptotic profile within the prefrontal cortex specifically. Building on this gap, the present study investigated whether LTG could mitigate LPS-induced working-memory impairment and whether this effect was associated with the concurrent modulation of these pathways within a single in vivo experimental framework. We hypothesized that LTG pretreatment would attenuate LPS-induced working-memory deficits by suppressing neuroinflammation, restoring redox homeostasis, and limiting apoptotic activation. Accordingly, working-memory performance was evaluated alongside prefrontal cortical biomarkers of inflammation, oxidative stress, and apoptosis. Thus, the novelty of this study lies in its integrated behavioral, molecular, and region-specific evaluation of whether the cognitive protection associated with LTG is accompanied by coordinated changes in these interconnected pathways (Figure 1).

## 2. Materials and Methods

### 2.1. Chemicals

High-grade analytical reagents were used throughout the study. LPS from *Escherichia coli* O111:B4 and LTG were obtained from Sigma-Aldrich (St. Louis, MO, USA). LTG was dispersed in a 0.5% *W*/*V* carboxymethylated cellulose polymer to ensure uniform oral dosing, whereas LPS was solubilized in 0.9% *W*/*V* NaCl just prior to administration. All biochemical parameters were measured using ready-to-use ELISA kits provided by MyBioSource (San Diego, CA, USA).

### 2.2. Experimental Animals

The experimental cohort consisted of 24 adult male Wistar rats, each weighing an average of 165 ± 15 g and aged 3 months. They were housed in a controlled environment at approximately 25 °C, with a consistent 12 h light/dark cycle. Rats were kept in groups of three per cage and had unlimited access to food and water at all times. Prior to the start of the experiments, a seven-day adaptation period was given to help the animals acclimate to their housing conditions. All procedures adhered to institutional ethical standards and received approval from the Committee of Research Ethics under the Deanship of Graduate Studies and Scientific Research at Qassim University (Approval No. 25-11-08).

Experimental procedures and reporting followed the ARRIVE 2.0 guidelines, including the Essential 10 recommendations. After the acclimation period, the rats were randomly assigned to four experimental groups (*n* = 6/group) to minimize selection bias. Group size was fixed in advance using an a priori power calculation run in G*Power (version 3.1; Heinrich Heine University Düsseldorf, Düsseldorf, Germany), informed by institutional animal-ethics guidance and effect sizes reported in our group’s earlier work). For the 2 × 2 factorial design, the calculation considered the LTG with LPS interaction, assuming a large effect size (*f* = 0.60), an α level of 0.05, and a statistical power (1 − β) of 0.80. The analysis indicated a minimum total sample size of 24 animals (*n* = 6/group), with spontaneous alternation percentage (SAP) in the Y-maze as the primary endpoint. Furthermore, behavioral assessments and biochemical analyses were performed by an investigator blinded to the treatment groups to ensure objective evaluation and reduce experimental bias.

### 2.3. Treatment Schedule

**Figure 2:**
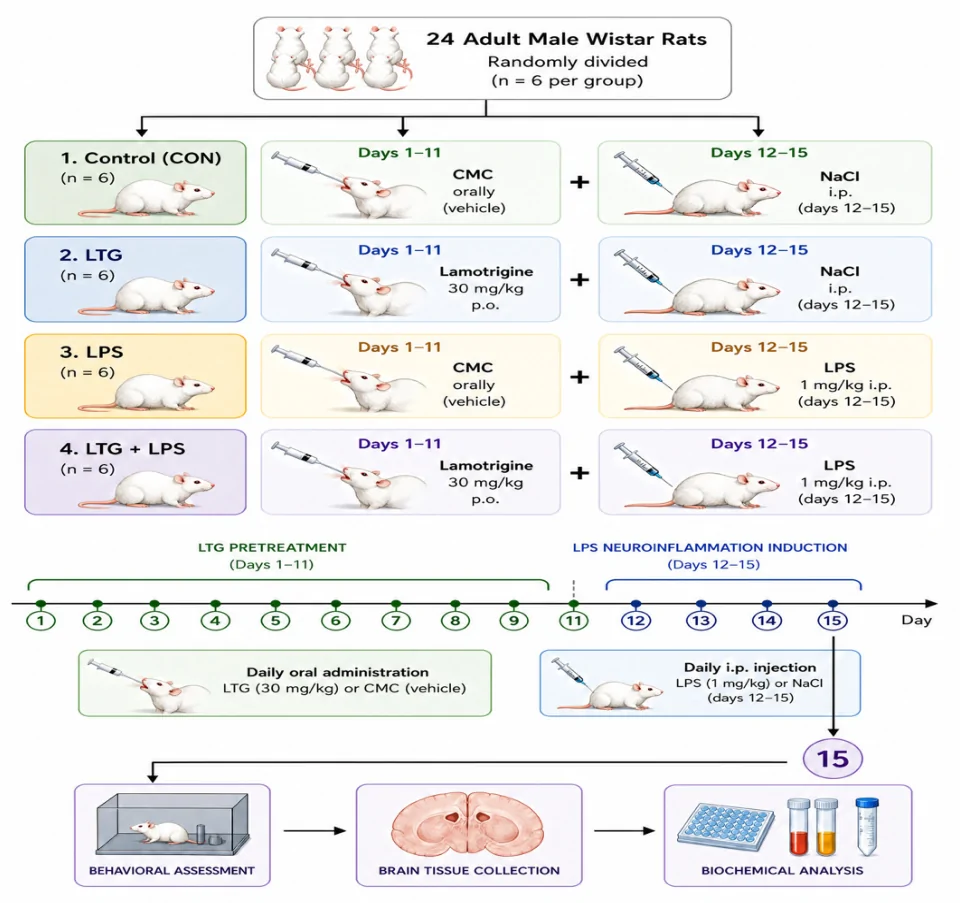
Experimental design and study workflow.

As outlined earlier, the animals were randomly grouped into four study arms (Figure 2). Group 1 (CON) served as the untreated control and received (0.5% *W*/*V* CMC) as a vehicle for 15 days, along with NaCl injections (0.9% *W*/*V*, 10 mL/kg, intraperitoneal) from days 12 to 15. Group 2 (LTG) was administered lamotrigine (30 mg/kg, p.o.) once on a daily cycle for 15 days and received NaCl injections following the same schedule as the control group. The LTG dose (30 mg/kg, p.o.) and 15-day treatment duration were selected based on prior experimental studies demonstrating its neuroprotective efficacy and safety in rodent models without inducing toxicity [34,35]. Group 3 (LPS) received the oral vehicle for 15 days and was administered LPS (1 mg/kg, i.p.) during the final four days (days 12–15) to induce neuroinflammation. Repeated administration of LPS (1 mg/kg, i.p.) for four consecutive days was used to establish a sub-acute neuroinflammation model, as previously described. This protocol reliably induces sustained neuroinflammatory responses and cognitive impairment, allowing evaluation of the neuroprotective effects of LTG pretreatment [25,31]. Group 4 (LTG + LPS) was treated with lamotrigine for 15 days and additionally received LPS injections on days 12–15.

### 2.4. Y-Maze Test

The Y-maze test was utilized as an assessment of spatial working memory, specifically through spontaneous alternation behavior in rats [37,38]. All testing took place between 09:00 and 13:00 h during the light cycle under stable environmental conditions, to keep circadian variation from confounding the memory measures. Animals were introduced into the central zone and permitted to freely explore all arms for 8 min. An entry was counted when the rat positioned all four paws within an arm. An alternation involved three consecutive visits to three distinct arms in sequence (e.g., ABC, BCA, CAB). Working memory performance was quantified as the SAP, computed using the formula: SAP (%) = [Number of actual alternations ÷ (Total arm entries − 2)] × 100. A higher SAP value indicates superior working memory, as animals with preserved memory characteristics tend to avoid re-entering an arm they have recently visited.

### 2.5. Preparation of Brain Homogenate

On the final experimental day, the Y-maze test was initiated 60 min after the final oral administration of LTG and lasted 8 min. Immediately after completion of the behavioral assessments, the rats were deeply anesthetized with ketamine (100 mg/kg, i.p.) and xylazine (10 mg/kg, i.p.). Thus, anesthesia was initiated approximately 68 min after the final LTG dose. Following confirmation of deep anesthesia, cervical dislocation was performed as the terminal procedure. Transcardial perfusion was not performed. The brain was removed within approximately 5–10 min after euthanasia, and the tissue was placed directly into ice-cold PBS (pH 7.4) and rinsed twice more to clear residual surface blood. The prefrontal cortex was then rapidly dissected on an ice-cold surface and immediately mechanically homogenized in ice-cold PBS (pH 7.4). The homogenate was centrifuged at 4000 rpm for 10 min, and the resulting supernatant was immediately collected and stored at −80 °C until biochemical analysis. All tissue samples were processed using the same standardized procedure to minimize variability related to post-mortem changes.

### 2.6. Bioassay of Neuroinflammatory Markers

The concentrations of COX-2, TNF-α, and IL-6 in the prefrontal cortex homogenates were determined using rat-specific ELISA kits supplied by MyBioSource (San Diego, CA, USA), following the manufacturer’s protocols. COX-2 was measured using Catalog No. 266603, which has a manufacturer-specified detection range of 0.156–10 ng/mL and a sensitivity of up to 0.05 ng/mL. TNF-α was analyzed using Catalog No. 162068, with a stated detection range of 5–100 ng/mL, whereas IL-6 was determined using Catalog No. 269892. Briefly, tissue samples were added to antibody-coated microplate wells and incubated with the corresponding detection reagents. After completion of the enzymatic color reaction, optical density was measured at 450 nm, and analytic concentrations were calculated from the respective standard curves.

### 2.7. Bioassay of Apoptotic Proteins

The concentrations of BCL-2, BAX, and Caspase-3 in the prefrontal cortex homogenates were determined using rat-specific ELISA kits according to the manufacturer’s instructions. BCL-2 was measured using Catalog No. 452319, with a detection range of 78.1–5000 pg/mL and a sensitivity of <30.98 pg/mL. BAX was analyzed using Catalog No. 2703209, which has a detection range of 0.312–20 ng/mL and a sensitivity of <0.125 ng/mL. Caspase-3 was measured using Catalog No. 261814, with a detection range of 1.56–100 ng/mL and a sensitivity of up to 0.5 ng/mL. Briefly, the tissue samples were added to antibody-coated microplate wells and incubated with the relevant detection reagents. Following completion of the colorimetric reaction, absorbance was measured at 450 nm, and the concentrations of the respective proteins were calculated using their standard curves.

### 2.8. Bioassay of Oxidative Stress Markers

MDA, GSH, and catalase levels in the prefrontal cortex homogenates were determined using rat-specific assay kits supplied by MyBioSource (San Diego, CA, USA), following the manufacturer’s instructions. MDA, an indicator of lipid peroxidation, was measured using Catalog No. 738685, with a detection range of 50–1000 ng/mL and a sensitivity of 1.0 ng/mL. GSH was determined using Catalog No. 2540412, which has a detection range of 2–100 µmol/L and a sensitivity of 2 µmol/L. Catalase was measured using Catalog No. 2704433, with a detection range of 0.78–50 ng/mL and a sensitivity of <0.30 ng/mL. Absorbance was recorded at the wavelength specified in the protocol for each kit, and the concentrations were calculated using the corresponding standard curves.

### 2.9. Statistical Analysis

Data are presented as mean ± SEM. Because the experimental design comprised two independent factors, LTG treatment (absent or present) and LPS exposure (absent or present), data were analyzed using two-way ANOVA to determine the main effects of LTG and LPS and the LTG with LPS interaction. Where appropriate, multiple comparisons were performed using Tukey’s post hoc test. Statistical analyses were performed using GraphPad Prism 9.0 (GraphPad Software, San Diego, CA, USA), and *p* < 0.05 was considered statistically significant.

## 3. Results

### 3.1. LTG Preserves Working Memory Performance in LPS-Challenged Rats

**Figure 3:**
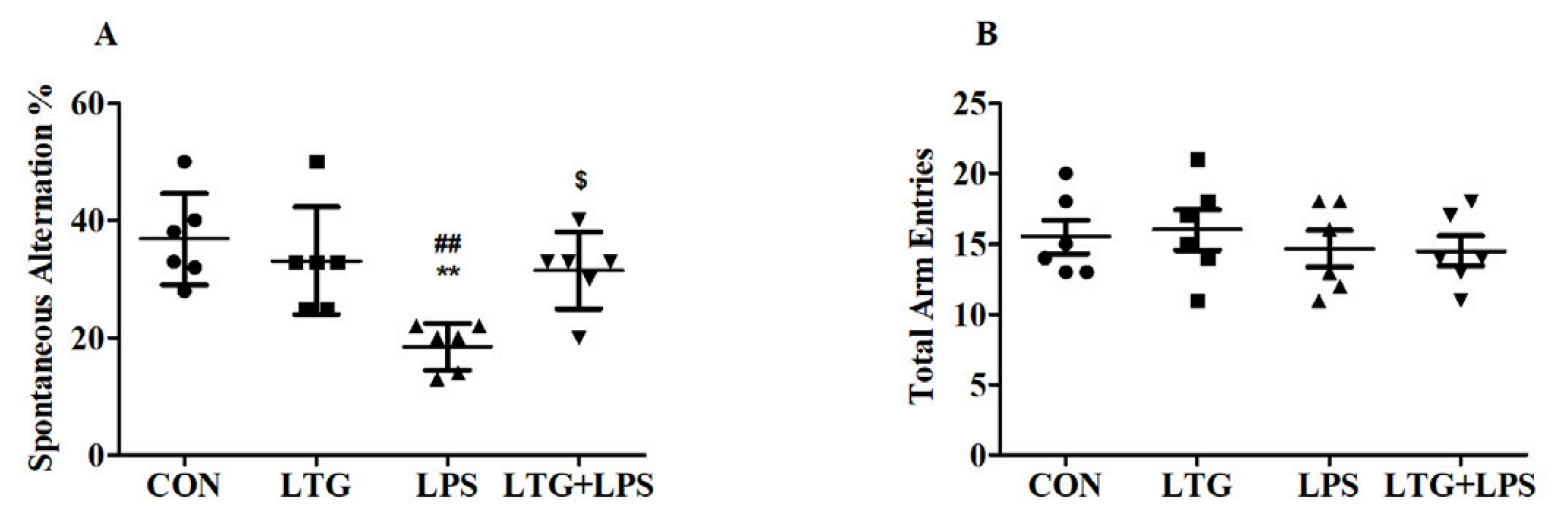
The effect of LTG on LPS-induced alterations in Y-maze performance is shown. (**A**) Spontaneous alternation percentage (SAP) and (**B**) total arm entries are presented. Data are expressed as mean ± SEM (*n* = 6/group) and were analyzed using two-way ANOVA with LTG treatment and LPS exposure as factors, followed by Tukey’s multiple-comparisons test. For SAP (**A**), two-way ANOVA revealed a significant main effect of LPS [F(1,20) = 11.87, *p* = 0.0026] and a significant LTG with LPS interaction [F(1,20) = 8.25, *p* = 0.0094], whereas the main effect of LTG was not significant [F(1,20) = 2.59, *p* = 0.1235]. For total arm entries (**B**), no significant main effects of LTG [F(1,20) = 0.018, *p* = 0.8938] or LPS [F(1,20) = 0.896, *p* = 0.3552] and no significant LTG with LPS interaction [F(1,20) = 0.073, *p* = 0.7896] were observed. Statistical notation for panel (**A**): ## *p* < 0.01 compared with CON; ** *p* < 0.01 compared with LTG; $ *p* < 0.05 compared with LPS.

The Y-maze spontaneous alternation test showed clear differences in working memory performance among the groups. Two-way ANOVA revealed a significant main effect of LPS [F(1,20) = 11.87, *p* = 0.0026] and a significant LTG with LPS interaction [F(1,20) = 8.25, *p* = 0.0094], whereas the main effect of LTG was not significant [F(1,20) = 2.59, *p* = 0.1235] (Figure 3A). Animals administered LPS demonstrated a significant reduction in SAP relative to the CON group (*p* < 0.01), indicating that LPS exposure was associated with impaired spatial working memory. Pretreatment with LTG (LTG + LPS group) substantially attenuated the LPS-induced deficit, as evidenced by a significant improvement in SAP relative to the LPS-only group (*p* < 0.05). Notably, LTG alone did not alter SAP relative to controls, suggesting that its effect may be protective against LPS-induced working-memory impairment rather than performance-enhancing in healthy animals.

Total arm entries did not significantly differ among the experimental groups (Figure 3B). Two-way ANOVA revealed no significant main effect of LTG [F(1,20) = 0.018, *p* = 0.8938] or LPS [F(1,20) = 0.896, *p* = 0.3552], and no significant LTG × LPS interaction [F(1,20) = 0.073, *p* = 0.7896]. These findings indicate that the observed alterations in spontaneous alternation performance were not accompanied by significant differences in overall arm-entry activity.

### 3.2. LTG Attenuated LPS-Induced Neuroinflammatory Response in Prefrontal Cortex

**Figure 4:**
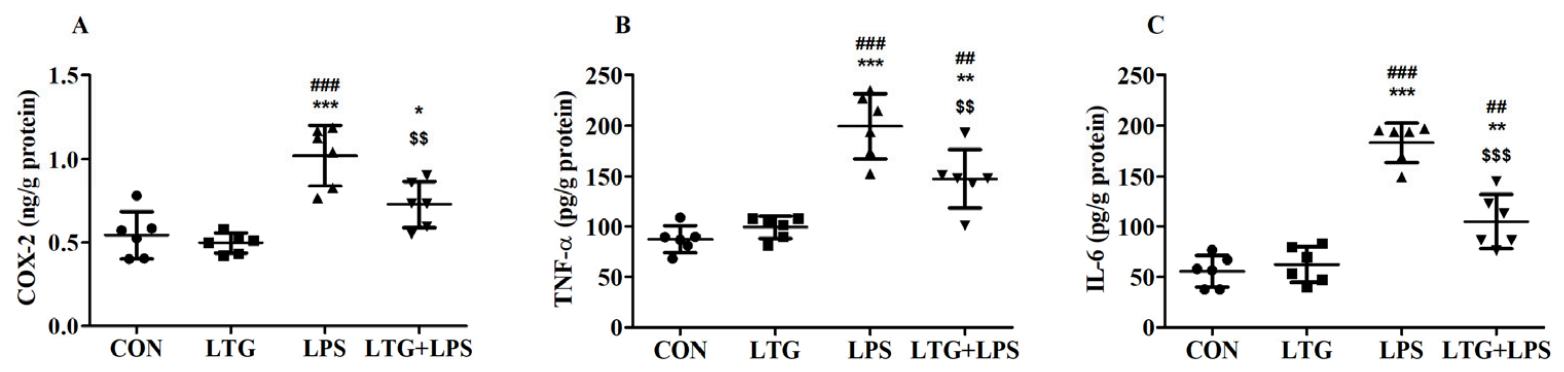
The effect of LTG on the LPS-induced increase in inflammatory mediators in the prefrontal cortex is shown. Concentrations of (**A**) COX-2, (**B**) TNF-α, and (**C**) IL-6 are presented. Data are expressed as mean ± SEM (*n* = 6/group) and were analyzed using two-way ANOVA with LTG treatment and LPS exposure as factors. For COX-2, significant main effects of LTG [F(1,20) = 9.04, *p* = 0.0070] and LPS [F(1,20) = 39.64, *p* < 0.0001] and a significant LTG with LPS interaction [F(1,20) = 4.79, *p* = 0.0407] were observed. For TNF-α, significant main effects of LTG [F(1,20) = 4.47, *p* = 0.0472] and LPS [F(1,20) = 71.30, *p* < 0.0001] and a significant LTG with LPS interaction [F(1,20) = 11.33, *p* = 0.0031] were observed. For IL-6, significant main effects of LTG [F(1,20) = 18.56, *p* = 0.0003] and LPS [F(1,20) = 105.04, *p* < 0.0001] and a significant LTG with LPS interaction [F(1,20) = 26.26, *p* < 0.0001] were observed. Multiple comparisons were performed using Tukey’s post hoc test. Statistical notation: ## *p* < 0.01, ### *p* < 0.001 compared with CON; * *p* < 0.05, ** *p* < 0.01, *** *p* < 0.001 compared with LTG; $$ *p* < 0.01, $$$ *p* < 0.001 compared with LPS.

Two-way ANOVA demonstrated significant main effects of LTG [F(1,20) = 9.04, *p* = 0.0070] and LPS [F(1,20) = 39.64, *p* < 0.0001] on COX-2 expression in the prefrontal cortex, with a significant LTG × LPS interaction [F(1,20) = 4.79, *p* = 0.0407] (Figure 4A). LPS exposure resulted in a significant increase in COX-2 levels compared with the CON group (*p* < 0.001), indicating a robust inflammatory reaction following endotoxin challenge. Pretreatment with LTG markedly counteracted this elevation in LTG + LPS-treated rats, producing a significant decline in COX-2 concentration compared with the LPS group (*p* < 0.01). These findings indicate that LTG attenuated the LPS-induced increase in COX-2 levels. LTG alone did not alter basal COX-2 expression, suggesting that its effects are more evident under inflammatory challenge.

TNF-α levels were markedly altered by the treatments. Two-way ANOVA revealed significant main effects of LTG [F(1,20) = 4.47, *p* = 0.0472] and LPS [F(1,20) = 71.30, *p* < 0.0001], together with a significant LTG with LPS interaction [F(1,20) = 11.33, *p* = 0.0031] (Figure 4B). LPS administration led to a marked elevation of TNF-α compared with the CON group (*p* < 0.001), consistent with an inflammatory response following LPS administration. LTG pretreatment significantly blunted the pro-inflammatory cytokine surge (*p* < 0.01 vs. LPS) in the LTG + LPS group, indicating attenuation of the LPS-induced inflammatory response in the prefrontal cortex. The LTG-only group did not differ significantly from controls, indicating that LTG does not interfere with normal cytokine homeostasis in the absence of inflammatory challenge.

IL-6 levels were also significantly affected by treatment. Two-way ANOVA revealed significant main effects of LTG [F(1,20) = 18.56, *p* = 0.0003] and LPS [F(1,20) = 105.04, *p* < 0.0001], together with a significant LTG with LPS interaction [F(1,20) = 26.26, *p* < 0.0001] (Figure 4C). LPS-treated rats exhibited a substantial increase in IL-6 expression compared to controls (*p* < 0.001), consistent with its role as a central mediator of neuroinflammatory amplification. LTG pretreatment in LTG + LPS rats significantly attenuated this LPS-induced IL-6 elevation (*p* < 0.001), indicating attenuation of the LPS-induced increase in IL-6 levels. Similar to other markers, LTG alone did not significantly modify baseline IL-6 levels, suggesting that its anti-inflammatory action is specifically engaged during pathological stimulation.

### 3.3. LTG Modulated Apoptosis-Associated Protein Expression

**Figure 5:**
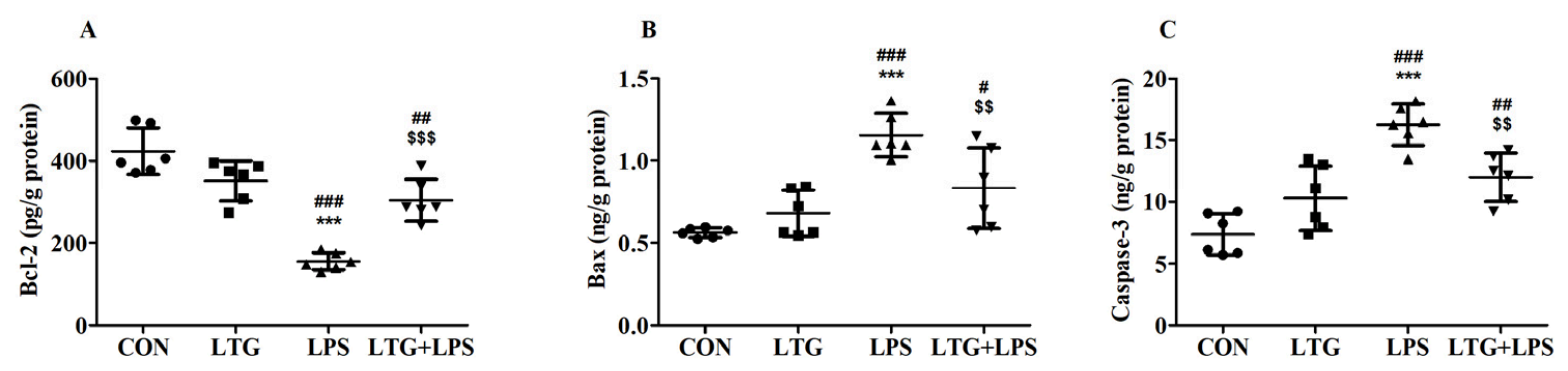
Modulatory effects of LTG on apoptosis-associated markers in rats treated with LPS are depicted. The levels of (**A**) Bcl-2, (**B**) BAX, and (**C**) Caspase-3 are presented. Values are reported as mean ± SEM (*n* = 6/group) and were analyzed using two-way ANOVA with LTG treatment and LPS exposure as factors. For Bcl-2, a significant main effect of LPS [F(1,20) = 68.93, *p* < 0.0001] and a significant LTG with LPS interaction [F(1,20) = 33.82, *p* < 0.0001] were observed, whereas the main effect of LTG was not significant [F(1,20) = 4.06, *p* = 0.0576]. For BAX, a significant main effect of LPS [F(1,20) = 34.01, *p* < 0.0001] and a significant LTG with LPS interaction [F(1,20) = 11.86, *p* = 0.0026] were observed, whereas the main effect of LTG was not significant [F(1,20) = 2.56, *p* = 0.1252]. For Caspase-3, a significant main effect of LPS [F(1,20) = 41.21, *p* < 0.0001] and a significant LTG with LPS interaction [F(1,20) = 19.05, *p* = 0.0003] were observed, whereas the main effect of LTG was not significant [F(1,20) = 0.65, *p* = 0.4286]. Multiple comparisons were performed using Tukey’s post hoc test. Statistical notation: # *p* < 0.05, ## *p* < 0.01, ### *p* < 0.001 compared with CON; *** *p* < 0.001 compared with LTG; $$ *p* < 0.01, $$$ *p* < 0.001 compared with LPS.

Two-way ANOVA revealed a significant main effect of LPS [F(1,20) = 68.93, *p* < 0.0001] and a significant LTG with LPS interaction [F(1,20) = 33.82, *p* < 0.0001] on Bcl-2 expression, whereas the main effect of LTG was not significant [F(1,20) = 4.06, *p* = 0.0576] (Figure 5A). LPS exposure significantly reduced Bcl-2 levels when compared with controls (*p* < 0.001), consistent with a reduction in the anti-apoptotic marker Bcl-2. Pretreatment with LTG markedly restored Bcl-2 expression (*p* < 0.001 vs. LPS) in the LTG + LPS treatment group, indicating attenuation of the LPS-induced reduction in Bcl-2 levels. LTG alone maintained Bcl-2 levels comparable to those in the control group, confirming that its regulatory effect emerges primarily under pathological conditions.

BAX levels were significantly altered by the treatments. Two-way ANOVA revealed a significant main effect of LPS [F(1,20) = 34.01, *p* < 0.0001] and a significant LTG with LPS interaction [F(1,20) = 11.86, *p* = 0.0026], whereas the main effect of LTG was not significant [F(1,20) = 2.56, *p* = 0.1252] (Figure 5B). LPS administration led to a significant increase in BAX expression compared with the control group (*p* < 0.001), consistent with increased apoptosis-related signaling following LPS exposure. LTG pretreatment significantly lowered BAX levels relative to the LPS group (*p* < 0.01) in LTG + LPS animals, consistent with attenuation of the LPS-induced increase in BAX levels. BAX expression in the LTG-only group remained comparable to controls, showing that LTG does not induce apoptotic stress in healthy tissue.

Caspase-3, a key executioner in apoptosis, also showed significant treatment differences. Two-way ANOVA revealed a significant main effect of LPS [F(1,20) = 41.21, *p* < 0.0001] and a significant LTG with LPS interaction [F(1,20) = 19.05, *p* = 0.0003], whereas the main effect of LTG was not significant [F(1,20) = 0.65, *p* = 0.4286] (Figure 5C). LPS produced a substantial elevation in active Caspase-3 compared with controls (*p* < 0.001), confirming downstream execution of apoptotic pathways. LTG pretreatment significantly reduced Caspase-3 levels (*p* < 0.01 vs. LPS) in LTG + LPS rats, indicating attenuation of the LPS-induced increase in active Caspase-3. LTG alone did not significantly alter Caspase-3 expression, further supporting its potential protective effect during inflammatory challenge.

### 3.4. LTG Lessened Oxidative Stress by Enhancing Antioxidant Activity

**Figure 6:**
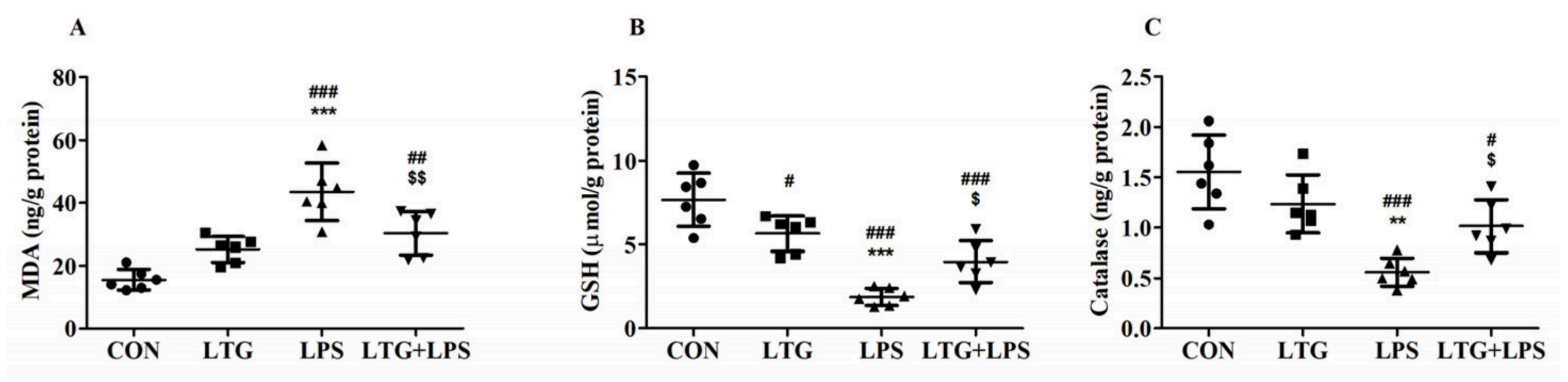
The impact of LTG on oxidative stress indices in the prefrontal cortex after LPS challenge is shown. The values of (**A**) MDA, (**B**) GSH, and (**C**) Catalase are displayed. Values are expressed as mean ± SEM (*n* = 6/group) and were analyzed using two-way ANOVA with LTG treatment and LPS exposure as factors. For MDA, a significant main effect of LPS [F(1,20) = 41.33, *p* < 0.0001] and a significant LTG with LPS interaction [F(1,20) = 19.71, *p* = 0.0003] were observed, whereas the main effect of LTG was not significant [F(1,20) = 0.50, *p* = 0.4897]. For GSH, a significant main effect of LPS [F(1,20) = 61.23, *p* < 0.0001] and a significant LTG with LPS interaction [F(1,20) = 18.61, *p* = 0.0003] were observed, whereas the main effect of LTG was not significant [F(1,20) = 0.008, *p* = 0.9281]. For Catalase, a significant main effect of LPS [F(1,20) = 28.84, *p* < 0.0001] and a significant LTG with LPS interaction [F(1,20) = 11.71, *p* = 0.0027] were observed, whereas the main effect of LTG was not significant [F(1,20) = 0.35, *p* = 0.5617]. Multiple comparisons were performed using Tukey’s post hoc test. Statistical notation: # *p* < 0.05, ## *p* < 0.01, ### *p* < 0.001 compared with CON; ** *p* < 0.01, *** *p* < 0.001 compared with LTG; $ *p* < 0.05, $$ *p* < 0.01 compared with LPS.

MDA levels were significantly affected by the treatments. Two-way ANOVA revealed a significant main effect of LPS [F(1,20) = 41.33, *p* < 0.0001] and a significant LTG with LPS interaction [F(1,20) = 19.71, *p* = 0.0003], whereas the main effect of LTG was not significant [F(1,20) = 0.50, *p* = 0.4897] (Figure 6A). LPS markedly increased MDA concentrations (*p* < 0.001), indicating enhanced lipid peroxidation and oxidative damage to membranes. In LTG + LPS animals, LTG pretreatment significantly attenuated this elevation (*p* < 0.01 vs. LPS), indicating attenuation of LPS-induced lipid peroxidation. LTG alone showed MDA levels comparable to the control group.

Treatment effects were also significant for GSH. Two-way ANOVA revealed a significant main effect of LPS [F(1,20) = 61.23, *p* < 0.0001] and a significant LTG with LPS interaction [F(1,20) = 18.61, *p* = 0.0003], whereas the main effect of LTG was not significant [F(1,20) = 0.008, *p* = 0.9281] (Figure 6B). LPS exposure led to a pronounced reduction in GSH (*p* < 0.001), reflecting depletion of endogenous antioxidant reserves. Rats (LTG + LPS) pretreated with LTG showed a significant restoration of GSH levels (*p* < 0.05 vs. LPS), indicating partial restoration of GSH levels following LPS exposure. The LTG-only group showed a modest but significant reduction in GSH compared with the control group (*p* < 0.05).

Catalase activity showed significant differences among groups. Two-way ANOVA revealed a significant main effect of LPS [F(1,20) = 28.84, *p* < 0.0001] and a significant LTG with LPS interaction [F(1,20) = 11.71, *p* = 0.0027], whereas the main effect of LTG was not significant [F(1,20) = 0.35, *p* = 0.5617] (Figure 6C). LPS challenge significantly reduced catalase levels compared with controls (*p* < 0.001), indicating compromised enzymatic antioxidant defense. LTG pretreatment in LTG + LPS rats significantly improved catalase activity (*p* < 0.05 vs. LPS), indicating partial restoration of catalase activity following LPS exposure. Catalase activity in LTG-only rats remained similar to controls, suggesting that LTG does not disrupt normal redox homeostasis.

## 4. Discussion

The current study suggests that LTG may attenuate LPS-induced working-memory impairment and associated inflammatory, oxidative-stress, and apoptosis-related changes in the rat prefrontal cortex. Using the Y-maze task, we observed that systemic LPS administration markedly reduced spontaneous alternation performance, indicating significant impairment of spatial working memory. This mirrors earlier work in which LPS was shown to interfere with hippocampal-prefrontal connectivity via microglial TLR4 activation, triggering cytokine release and synaptic impairment [24,39]. Importantly, LTG pretreatment attenuated these LPS-evoked deficits, with working memory performance approaching control levels. Consistent with our findings, Nowakowska et al. previously demonstrated that repeated LTG administration improved spatial memory in rats assessed using the Morris water maze under both standard and enriched environmental conditions [40]. Therefore, the improvement observed in the present study should not be considered the first evidence of a memory-enhancing effect of LTG; rather, it extends previous evidence by demonstrating improved working memory in an LPS-induced neuroinflammation model. The present study further extends these findings by linking LTG-associated improvement in working memory with coordinated changes in inflammatory, oxidative-stress, and apoptotic pathways in the prefrontal cortex. Thus, the novelty lies in the integrated behavioral, molecular, and region-specific evaluation rather than in proposing entirely new individual pharmacological actions of LTG. Similar beneficial effects of LTG on cognitive performance have been described in other neuroinflammatory and excitotoxic models, highlighting its ability to stabilize glutamatergic transmission and reduce neural hyper-excitability [27,41].

A growing body of evidence points to neuroinflammation as a central driver of toxic injury [42]. Neuroinflammatory profiling further confirmed LPS’s detrimental impact on prefrontal cortical tissue. LPS-challenged animals exhibited significant upregulation of IL-6, COX-2, and TNF-α, consistent with TLR4-mediated activation of downstream inflammatory cascades and transcriptional upregulation of cytokine networks. Elevated COX-2 and pro-inflammatory cytokine levels are well-established hallmarks of LPS-induced neuroinflammation and have been strongly linked to synaptic impairment, neuronal injury, and cognitive decline [43,44]. In parallel, toxicant exposure can promote reactive oxygen species generation, increase oxidative burden, and contribute to cellular damage [45]. LTG pretreatment significantly attenuated these increases across all inflammatory markers, suggesting an attenuation of the inflammatory response. Previous studies similarly reported that LTG reduces IL-6, TNF-α, and COX-2 production in both in vitro and in vivo inflammation models [32,46], supporting our observations and suggesting that LTG’s anti-inflammatory activity may contribute to its protective effects under inflammatory conditions.

Programmed cell death pathways are activated in response to various toxicant-induced injuries [47,48]. Caspase-3 is a key executioner protease in apoptosis, and its activation in neural tissue indicates the involvement of programmed cell death in response to severe cellular stress [49,50]. The balance between pro-apoptotic BAX and anti-apoptotic Bcl-2 proteins serves as an important indicator of cellular fate and apoptotic regulation [51]. Collectively, Caspase-3, Bcl-2, and BAX serve as complementary biomarkers for assessing neurotoxic effects and neuroprotective responses [1]. The LPS-induced inflammatory environment was accompanied by pronounced pro-apoptotic signaling, characterized by reduced Bcl-2 and increased BAX and Caspase-3 levels. These alterations are consistent with the activation of apoptosis-related processes commonly associated with neuroinflammatory injury and neuronal dysfunction [52,53]. Neuroinflammation driven by activated microglia and reactive astrocytes can further amplify neuronal injury through the release of pro-inflammatory mediators, thereby contributing to synaptic dysfunction and neuronal loss [54]. LTG pretreatment attenuated these LPS-induced apoptotic changes, as reflected by increased Bcl-2 and reduced BAX and Caspase-3 levels. This aligns with earlier findings demonstrating that LTG inhibits mitochondrial apoptotic pathways, reduces cytochrome c release, and suppresses executioner caspase activity under inflammatory or excitotoxic conditions [30,55]. Collectively, these findings suggest that LTG may exert protective effects by attenuating apoptosis-associated signaling during LPS-induced neuroinflammation.

Oxidative stress is widely recognized as a key contributor to neurotoxicity [56]. An imbalance between reactive oxygen species and antioxidant defenses leads to the accumulation of oxidative damage products, including lipid peroxidation, protein oxidation, and oxidized nucleotides [57]. Markers such as MDA, 4-hydroxynonenal, isoprostanes, and GSH ratios provide useful indicators of lipid oxidative damage and altered antioxidant capacity, while also reflecting mechanisms linking oxidative stress to inflammation and apoptosis [1]. Oxidative stress is another critical pathological process linked to LPS-induced neurotoxicity. Our findings indicated that LPS significantly elevated MDA levels, indicating lipid peroxidation, while simultaneously depleting antioxidant defenses, including GSH and catalase. These alterations reflect a shift toward a pro-oxidant state that compromises neuronal integrity and accelerates neurodegeneration [58,59]. LTG pretreatment significantly attenuated these LPS-induced alterations by reducing MDA and normalizing antioxidant defenses, suggesting an improvement in endogenous antioxidant defenses. Earlier research supports these findings, showing that LTG elevates antioxidant enzyme activity, reduces free radical accumulation, and prevents oxidative membrane damage in models of ischemia, depression, and inflammation [33,36].

Taken together, these findings indicate that LTG concurrently modulates inflammatory, oxidative, and apoptosis-related pathways within the same experimental system, and that these changes are accompanied by preserved working-memory performance following LPS challenge. We acknowledge that the individual anti-inflammatory, antioxidant, and anti-apoptotic effects of LTG observed in the present study have been reported previously in other experimental models [27,29]. Therefore, the contribution of the present study does not lie in demonstrating these individual pharmacological effects for the first time, but rather in examining their concurrent modulation in the specific context of LPS-induced neuroinflammation and working-memory impairment. By assessing these interconnected pathways together with a behavioral outcome in the same experimental model, the present findings extend previous evidence and provide an integrated view of the potential neuroprotective effects of LTG against inflammation-associated working-memory impairment. Given the involvement of neuroinflammation and oxidative injury in numerous CNS disorders, these findings provide a rationale for further investigation of the potential preventive and protective effects of LTG in experimental models involving neuroinflammation and oxidative injury.

Despite the promising findings, several limitations should be acknowledged. A limitation of this work is that we relied on a single behavioral readout, the Y-maze alternation test, which taps mainly working memory; inclusion of additional paradigms would provide a broader cognitive evaluation. Furthermore, the biochemical analyses were restricted to the prefrontal cortex, and assessing additional regions, such as the hippocampus, would enhance understanding of the overall neurobiological effects of LTG. Moreover, the biochemical measurements were obtained from prefrontal cortex tissue homogenates and therefore could not identify the specific cellular sources of the observed changes or directly demonstrate neuronal survival or apoptosis. Transcardial perfusion was also not performed; therefore, although the isolated brains were washed with ice-cold PBS and rapidly processed under standardized conditions, residual intravascular blood and post-mortem changes may have influenced some biochemical measurements. Additionally, pharmacokinetic analyses of LTG were not performed; therefore, plasma and brain concentrations of LTG could not be determined, limiting our ability to directly relate the administered dose to systemic and central drug exposure. Furthermore, the present findings demonstrate associations between behavioral improvement and molecular changes but do not establish direct causal relationships among the inflammatory, oxidative-stress, and apoptotic pathways. Finally, the study employed a sub-acute LPS model and evaluated only a single LTG dose. Future studies incorporating additional cognitive tests, pharmacokinetic assessments, pathway-specific interventions, long-term experimental models, and dose–response analyses are warranted to further characterize the mechanisms and potential neuroprotective effects of LTG.

## 5. Conclusions

Overall, this study suggests that LTG may attenuate LPS-induced working memory deficits, accompanied by modulation of inflammatory, oxidative, and apoptosis-associated markers in the prefrontal cortex. LPS challenge increased COX-2, TNF-α, IL-6, lipid peroxidation, and pro-apoptotic markers, while LTG pretreatment attenuated these changes. These findings suggest that the protective effects of LTG pretreatment against LPS-induced working-memory impairment are associated with changes in inflammatory, oxidative, and apoptosis-related markers. Further studies incorporating additional cognitive paradigms, brain regions, doses, and longer treatment periods are warranted to better characterize the effects of LTG and explore its potential preventive and protective effects against neuroinflammation-associated working-memory impairment.

## Data Availability

The data presented in this study are available in the article; further inquiries can be directed to the corresponding author.

## Abbreviations

The following abbreviations are used in this manuscript:

AD	Alzheimer’s disease
BAX	BCL-2-associated X protein
BCL-2	B-cell lymphoma 2
CNS	Central nervous system
CON	Control
COX-2	Cyclooxygenase-2
ELISA	Enzyme-linked immunosorbent assay
GSH	Glutathione
HRP	Horseradish peroxidase
IL-1	Interleukin-1
IL-6	Interleukin-6
LPS	Lipopolysaccharide
LTG	Lamotrigine
MDA	Malondialdehyde
NaCl	Sodium chloride
PBS	Phosphate-buffered saline
ROS	Reactive oxygen species
SAP	Spontaneous alternation percentage
SOD	Superoxide dismutase
TLR4	Toll-like receptor 4
TNF-α	Tumor necrosis factor-alpha
